# High Lethality of *Mycobacterium tuberculosis* Infection in Mice Lacking the Phagocyte Oxidase and Caspase1/11

**DOI:** 10.1128/iai.00060-23

**Published:** 2023-06-14

**Authors:** Sean M. Thomas, Andrew J. Olive

**Affiliations:** a Department of Microbiology and Molecular Genetics, College of Osteopathic Medicine, Michigan State University, East Lansing, Michigan, USA; Weill Cornell Medicine

**Keywords:** Caspase1, genetic interactions, *Mycobacterium tuberculosis*, NADPH phagocyte oxidase, inflammasome

## Abstract

Immune networks that control antimicrobial and inflammatory mechanisms have overlapping regulation and functions to ensure effective host responses. Genetic interaction studies of immune pathways that compare host responses in single and combined knockout backgrounds are a useful tool to identify new mechanisms of immune control during infection. For disease caused by pulmonary Mycobacterium tuberculosis (Mtb) infections, which currently lacks an effective vaccine, understanding the genetic interactions between protective immune pathways may identify new therapeutic targets or disease-associated genes. Previous studies have suggested a direct link between the activation of NLRP3-Caspase1 inflammasome and the NADPH-dependent phagocyte oxidase complex during Mtb infection. Loss of the phagocyte oxidase complex alone resulted in increased activation of Caspase1 and IL-1β production during Mtb infection, resulting in failed disease tolerance during the chronic stages of disease. To better understand this interaction, we generated mice lacking both *Cybb*, a key subunit of the phagocyte oxidase, and *Caspase1/11*. We found that *ex vivo* Mtb infection of *Cybb^−/−^Caspase1/11^−/−^* macrophages resulted in the expected loss of IL-1β secretion but an unexpected change in other inflammatory cytokines and bacterial control. Mtb infected *Cybb^−/−^Caspase1/11^−/−^* mice rapidly progressed to severe TB, succumbing within 4 weeks to disease characterized by high bacterial burden, increased inflammatory cytokines, and the recruitment of granulocytes that associated with Mtb in the lungs. These results uncover a key genetic interaction between the phagocyte oxidase complex and Caspase1/11 that controls protection against TB and highlight the need for a better understanding of the regulation of fundamental immune networks during Mtb infection.

## INTRODUCTION

Defense against infection requires the regulated activation of immune networks that determine the magnitude and duration of the host response (1, 2). Dysregulation of these immune networks contributes to increased susceptibility to infection and reduced disease tolerance (3, 4). During lung infections with Mycobacterium tuberculosis, pro-inflammatory responses mediated by cytokines such as interleukin-1 beta (IL-1β), tumor necrosis factor (TNF), and interferon gamma (IFN-γ) must be strong enough to restrict infection, while maintaining respiratory function and controlling tissue damage (5, 6). This balance is controlled by the tight regulation of cytokine and chemokine secretion to effectively direct the inflammatory process and immune cell recruitment (7, 8). Disruption of this balance contributes to progressive inflammatory tuberculosis (TB) disease, which causes over 1.5 million deaths each year (9). Understanding the factors contributing to protection or susceptibility during Mtb infection is essential to devise more effective therapies and immunization strategies.

TB susceptibility is controlled by a combination of bacterial, host, and environmental factors (10, 11). Many defined protective host genes comprise the Mendelian susceptibility to mycobacterial diseases (MSMD) (12). Patients with these conditions have loss-of-function alleles in genes that are essential for protective host responses such as IFN-γ signaling. Additional genes related to autophagy, reactive oxygen and nitrogen species (ROS/RNS) production, and cytokine production are also protective in the mouse model of Mtb (13, 14). However, while many genes are now identified as protective against Mtb, the precise mechanisms by which they control disease remains unclear.

One such protective mechanism is the ROS produced by the NADPH phagocyte oxidase (15). In humans, chronic granulomatous disease (CGD) in patients with dysfunctional phagocyte oxidase complexes is associated with increased susceptibility to mycobacterial infections (16). Mice deficient in the phagocyte oxidase subunit *Cybb* control Mtb replication yet show defects in disease tolerance that result in a modest reduction in survival following high-dose Mtb infection (17, 18). The loss of *Cybb* results in the hyperactivation of the NLRP3 inflammasome and exacerbated IL-1β production by bone marrow-derived macrophages (BMDMs) and *in vivo* during murine Mtb infection (17). This exacerbated IL-1β can be reversed in BMDMs with chemical inhibitors of NLRP3 or Caspase1. Caspase1 is a critical component of the NLRP3 inflammasome and is responsible for the activation of mature IL-1β, IL-18, and gasdermin D (19). However, while Mtb infection of Caspase1-deficient macrophages results in loss of mature IL-1β production, mice lacking Caspase1/11 have no defects in IL-1β and minimal changes in susceptibility to TB *in vivo* (20). Even though previous studies have found clear links between phagocyte oxidase and the NLRP3 inflammasome that contribute to protection during Mtb infection, how these pathways interact and regulate each other’s function remains unclear.

Here, we used a genetic approach to understand interactions between the phagocyte oxidase and the inflammasome by generating *Cybb^−/−^Caspase1/11^−/−^* animals. Mtb infection of macrophages and dendritic cells from these animals reversed the exacerbated IL-1β production that was responsible for failed tolerance in *Cybb*^−/−^ cells. However, we found dysregulation of other pro-inflammatory mediators and reduced bacterial control during infection of *Cybb^−/−^Caspase1/11^−/−^* BMDMs. *In vivo*, we uncovered a synthetic susceptibility with *Cybb^−/−^Caspase1/11^−/−^* animals succumbing rapidly to TB disease within 4 weeks. We observed the loss of bacterial control and the recruitment of permissive granulocytes in *Cybb^−/−^Caspase1/11^−/−^* that were not seen in wild-type, *Cybb^−/−^* or *Caspase1/11^−/−^* animals. Thus, our results uncovered a previously unknown genetic interaction between the phagocyte oxidase and the Caspase1 inflammasome that contributes to TB protection. Furthermore, our results highlight the complexity of the interactions between immune networks that control Mtb susceptibility and the importance of the regulation of inflammatory cytokines in the lung environment.

## RESULTS

### Loss of Caspase 1/11 results in decreased IL-1β production in *Cybb^−/−^* phagocytes.

**FIG 1:**
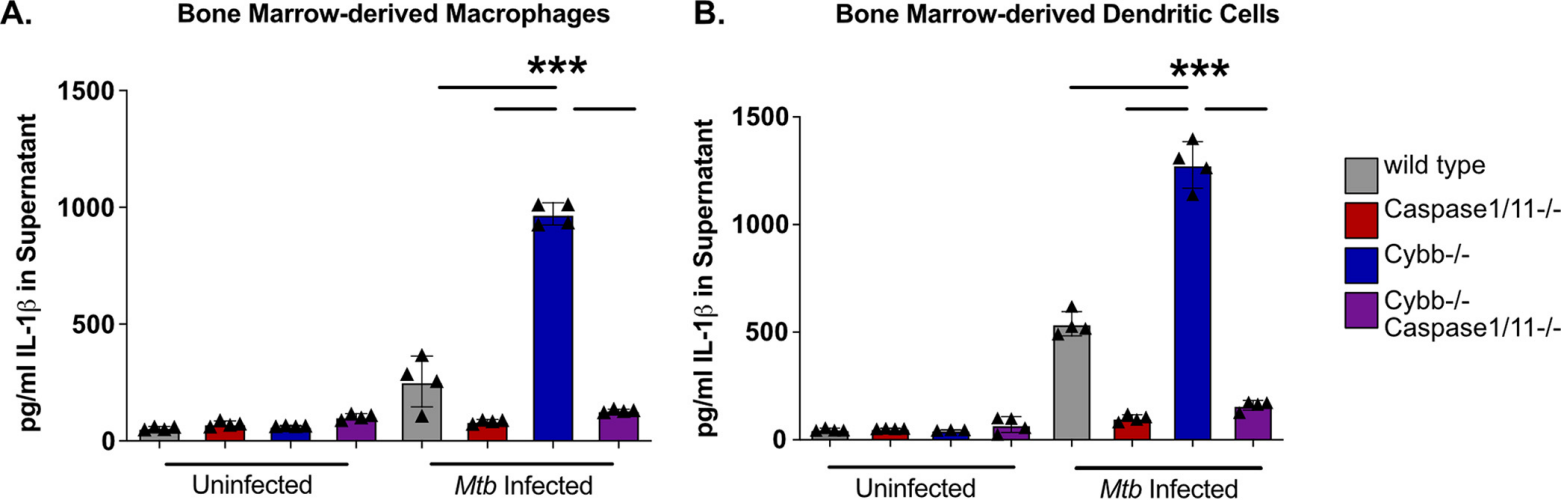
Exacerbated IL-1β following Mtb infection of *Cybb^−/−^* myeloid-cells is dependent on Caspase1/11. (A) BMDMs or (B) BMDCs from wild-type, *Caspase1/11^−/−^*, *Cybb^−/−^*, and *Cybb^−/−^Caspase1/11^−/−^* mice were left uninfected or infected with Mtb H37Rv at an MOI of 5. 14 h later, IL-1β was quantified from the supernatants by ELISA. Each point represents data from a single well from one representative experiment of three similar experiments. ***, *P* < 0.001 by one-way analysis of variance (ANOVA) with Tukey’s test for multiple comparisons.

Macrophages deficient in the phagocyte oxidase subunit *Cybb* produce elevated levels of IL-1β during Mtb infection (17). We previously showed this increased IL-1β is not due to changes in transcription but rather due to a hyper-active NLRP3 inflammasome that drives increased processing of Pro-IL-1β to mature IL-1β. To better understand the genetic interaction between Cybb and the inflammasome we generated mice deficient in both *Cybb* and *Caspase1/11* in the C57BL6/J background. We found that *Cybb^−/−^Caspase1/11^−/−^* mice were viable, with no changes in litter size or sex distribution and normal mendelian ratios. Over the course of our experiments, we noticed no changes in mouse behavior or development other than the progression of joint disease previously observed in *Cybb^−/−^* mice (17, 21). Cytokine analysis of the serum from uninfected animals found no significant differences between genotypes, suggesting that these mice are not hyper-inflammatory at baseline conditions (Fig. S1 in the supplemental material). To understand how the loss of *Cybb* and *Caspase1/11* altered the response to Mtb infection, we first examined the release of IL-1β during Mtb infection in *Cybb^–/–^*, *Caspase1/11^–/–^*, and *Cybb^−/−^Caspase1/11^−/−^* cells. Since Caspase1 is required for IL-1β activation we hypothesized that cells lacking *Caspase1/11* or both *Cybb* and *Caspase1/11* would be unable to secrete IL-1β following infection. To test this hypothesis, bone marrow-derived macrophages (BMDMs) from wild-type, *Cybb^−/−^*, *Caspase1/11^−/−^*, and *Cybb^−/−^Caspase1/11^−/−^* mice were infected with Mtb H37Rv. At 14 h later, the supernatants were removed from infected and uninfected control cells and the levels of IL-1β were quantified by enzyme-linked immunosorbent assay (ELISA). We observed no IL-1β release from uninfected cells and, in alignment with previous studies, we found that Mtb-infected *Cybb^−/−^* phagocytes secreted significantly more IL-1β compared to wild-type cells while *Caspase1/11^−/−^* cells released nearly undetectable levels of IL-1β (Fig. 1A) (17). Loss of Caspase1/11 in combination with Cybb resulted in no IL-1β release, similar to what was observed in *Caspase1/11^−/−^* macrophages. The experiment was repeated using bone marrow-derived dendritic cells (BMDCs) and the results were consistent with BMDMs. *Cybb^−/−^* cells produce high levels of IL-1β, which is reversed in the absence of Caspase1/11 (Fig. 1B). These data show that loss of Caspase1/11 reverses the elevated IL-1β production observed in *Cybb*-deficient phagocytes infected with Mtb.

### *Cybb^−/−^Caspase1/11^−/−^* BMDMs dysregulate cytokines and Mtb control during infection.

**FIG 2:**
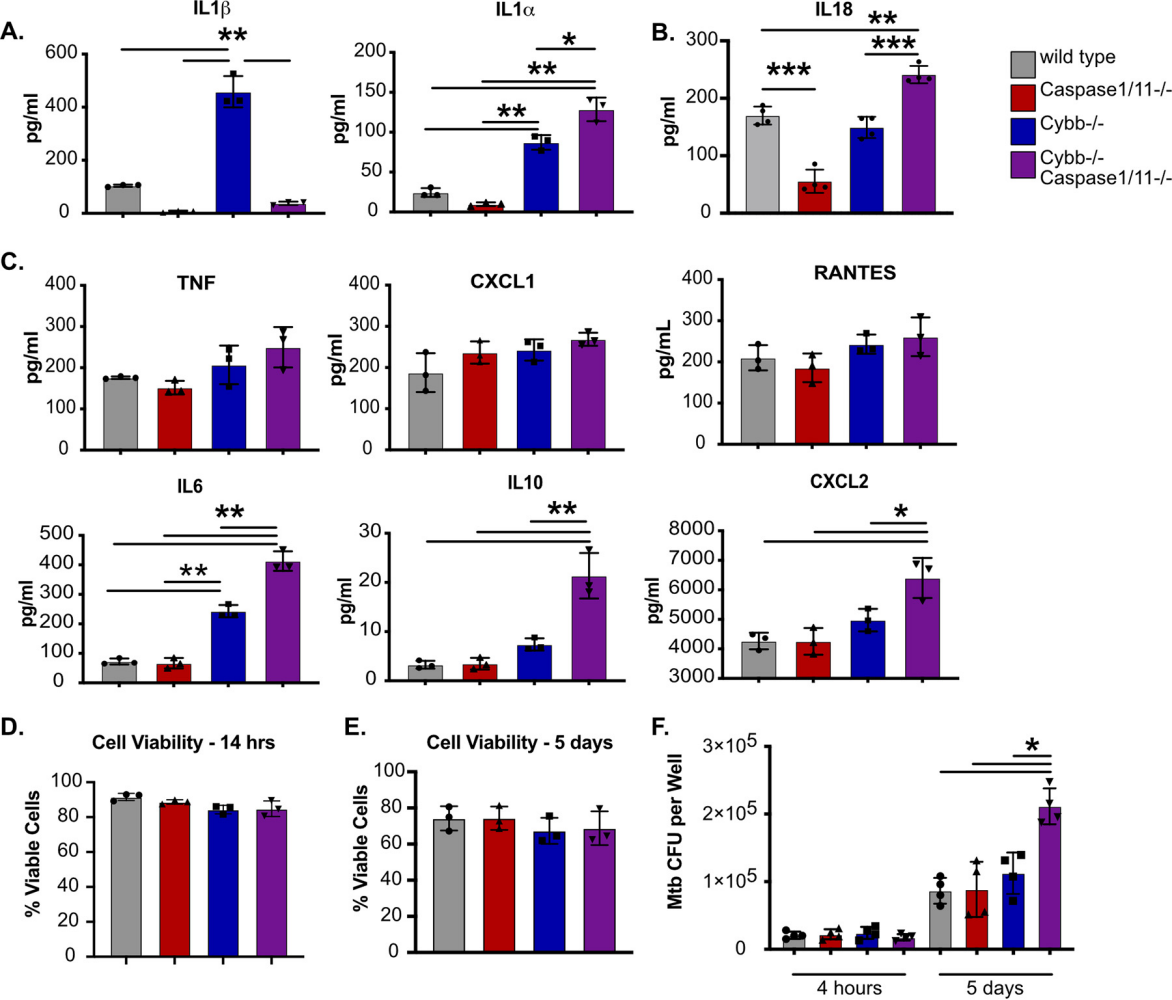
*Cybb^−/−^Caspase1/11^−/−^* macrophages are hyperinflammatory and permissive to bacterial growth during Mtb infection. (A) BMDMs from wild type, *Caspase1/11^−/−^*, *Cybb^−/−^*, and *Cybb^−/−^Caspase1/11^−/−^* mice were infected with Mtb H37Rv at an MOI of 5. 14 h later, IL-1β and IL-1α were quantified in the supernatant by Luminex multiplex assay and (B) IL-18 was quantified by ELISA. (C) BMDMs from wild type, *Caspase1/11^−/−^*, *Cybb^−/−^*, and *Cybb^−/−^Caspase1/11^−/−^* mice were infected with Mtb H37Rv at an MOI of 5. Levels of the indicated cytokines (TNF, CXCL1, RANTES, IL-6, IL-10, and CXCL2) were measured the following day. (D) BMDMs from wild type, *Caspase1/11^−/−^*, *Cybb^−/−^* and *Cybb^−/−^Caspase1/11^−/−^* mice were infected with Mtb H37Rv at an MOI of 5 or (E) an MOI of 1. At the indicated time points (14 h or 5 days), viable cells were were quantified. (F) BMDMs from wild type, *Caspase1/11^−/−^*, *Cybb^−/−^*, and *Cybb^−/−^Caspase1/11^−/−^* mice were infected with Mtb H37Rv at an MOI of 1. At the indicated time points, cells were lysed and viable Mtb CFU were quantified. In all experiments, each point represents data from a single well and shown is mean ± standard deviation (SD) from one experiment representative of two or three similar experiments. *, *P* < 0.05; **, *P* < 0.01; NS, not significant by one-way ANOVA with a Tukey’s test for multiple comparisons. All comparisons between samples were made. No indicator of significance indicates that the comparison was deemed “not significant.”

Both the ROS produced by the phagocyte oxidase and the immune pathways regulated by the inflammasome can modulate the inflammatory state of macrophages (22, 23). To better understand how the functions of *Cybb* and *Caspase1/11* interact to regulate inflammation, we infected BMDMs from each genotype with Mtb for 14 h and quantified cytokine release via multiplex cytokine analysis. Similar to the ELISA above, we observed increased IL-1β production by *Cybb^−/−^* macrophages which was reversed in macrophages from *Cybb^−/−^Caspase1/11^−/−^* mice (Fig. 2B). While IL-1α production was also increased by Cybb^−/−^ cells, this was not reversed and was, in contrast to IL-1β, exacerbated in *Cybb^−/−^Caspase1/11^−/−^* macrophages. The increased IL-1α production was not due to loss of Caspase1/11 alone, since *Caspase1/11^−/−^* BMDMs produced nearly undetectable levels of IL-1α following Mtb infection. We also examined the production of IL-18 by ELISA that was not in the multiplex panel but is a cytokine that is regulated by Caspase1 activation (24, 25). While we did not observe robust induction of IL-18 across any conditions, we did note a decrease in IL-18 production in cells lacking Caspase1/11, and a small but significant increase in IL-18 production in cells lacking Caspase1/11 and Cybb (Fig. 2C). Thus, IL-1α and IL-18 production by BMDMs is dysregulated in the absence of both *Cybb* and *Caspase1/11*.

The multiplex cytokine panel included a range of other inflammatory cytokines that were compared between each macrophage genotype (Fig. 2C). Most cytokines, including TNF, RANTES, and CXCL1, showed no significant difference between any of the genotypes. However, IL-6 and IL-10 production were both significantly increased by *Cybb^−/−^* BMDMs which was further exacerbated by *Cybb^−/−^Caspase1/11^−/−^* cells. Finally, CXCL2 was significantly increased only in *Cybb^−/−^Caspase1/11^−/−^* macrophages. Importantly, we observed no differences in cytokine production across all genotypes from uninfected cells, suggesting that cytokine differences are dependent on macrophage activation (Fig. S2). Taken together, *Cybb^−/−^Caspase1/11^−/−^* macrophages dysregulate a range of inflammatory cytokines in response to Mtb infection.

Since the inflammatory milieu was altered during infection of *Cybb^−/−^Caspase1/11^−/−^* BMDMs, we next tested whether intracellular control of Mtb growth was compromised. We infected BMDMs from each genotype with Mtb and measured Mtb growth and cell death. We observed no significant difference between genotypes in bacterial uptake 4 h following infection (Fig. 2D) or cell death 14 h or 5 days following infection. When we quantified CFU 5 days following infection, we observed no change in bacterial control in *Cybb^−/−^* or *Caspase1/11^−/−^* BMDMs but found significantly more Mtb growth in *Cybb^−/−^Caspase1/11^−/−^* BMDMs. These data suggest that the loss of *Cybb* and *Caspase1/11* together does not compromise cell survival but does result in less effective Mtb control and dysregulated cytokine production that does not occur in either knockout mouse genotype alone.

### *Cybb^−/−^Caspase1/11^−/−^* mice are hyper-susceptible to Mtb infection.

**FIG 3:**
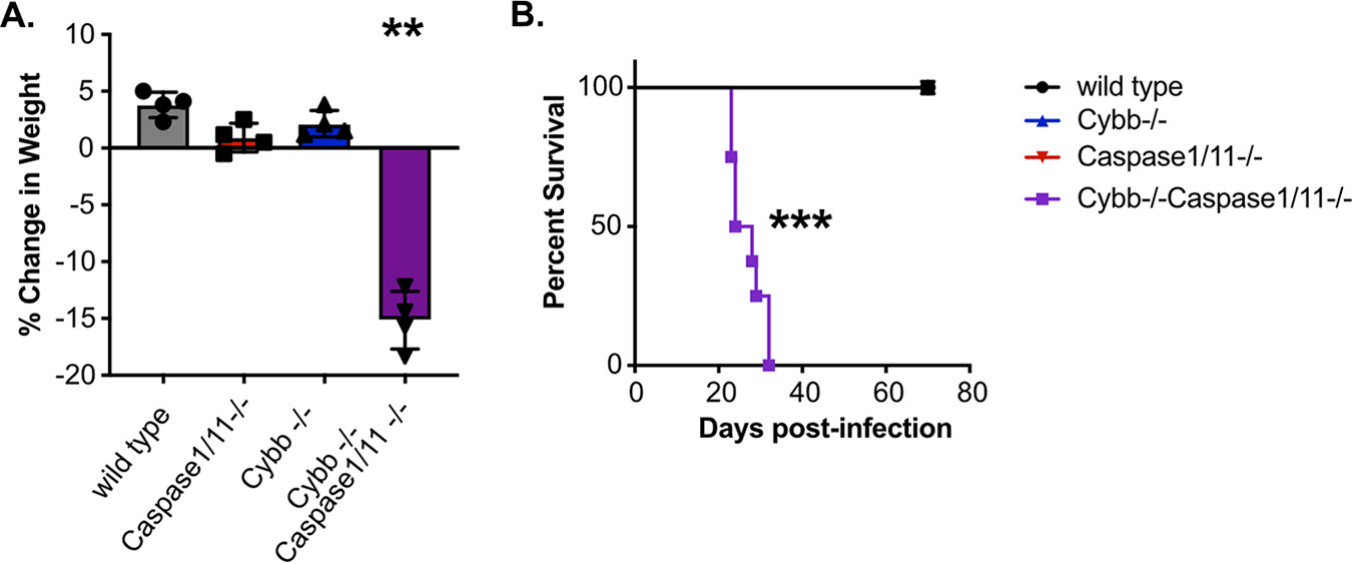
*Cybb^−/−^Caspase1/11^−/−^* mice rapidly succumb to pulmonary Mtb infection. Wild-type, *Caspase1/11^−/−^*, *Cybb^−/−^*, and *Cybb^−/−^Caspase1/11^−/−^* mice were infected with Mtb H37Rv by the aerosol route in a single batch (day 1: 50 to 150 CFU). (A) Changes in mouse weight from day 0 to 24 days postinfection were quantified. Data are from one experiment and are representative of three similar experiments with at least 4 mice per group. Statistics were determined by a Mann-Whitney test; **, *P* < 0.01. (B) The relative survival of each genotype was quantified over 75 days of infection. Data are pooled from two independent experiments (8 mice per group total). Statistics were determined by a Mantel-Cox test; ***, *P* < 0.001.

Our experiments in BMDMs showed that the loss of *Cybb* and *Caspase1/11* together results in dysregulated host responses during Mtb infection. We hypothesized that this dysregulation would result in changes to *in vivo* TB disease progression. To test this hypothesis, wild type, *Cybb^−/−^*, *Caspase1/11^−/−^*, and *Cybb^−/−^Caspase1/11^−/−^* mice were infected with Mtb by low-dose aerosol. As mice were monitored during the infection, we observed dramatic weight loss in *Cybb^−/−^Caspase1/11^−/−^* animals that required almost all of them to be euthanized prior to 30 days postinfection (Fig. 3A). In contrast, all other genotypes had gained weight over the same duration of infection. Survival analysis during these infections found that *Cybb^−/−^Caspase1/11^−/−^* mice are highly susceptible to Mtb infection, with all animals requiring euthanasia earlier than 5 weeks postinfection (Fig. 3B). In contrast, wild type, *Cybb^−/−^*, and *Caspase1/11^−/−^* animals all survived beyond day 75, similar to previous studies (20, 26). This observation suggests a strong genetic interaction between *Cybb* and *Caspase1/11* that results in the synthetic hyper-susceptibility of animals to *Mtb* infection.

### Mtb infection of *Cybb^−/−^Caspase1/11^−/−^* mice results in increased bacterial growth and inflammatory cytokine production.

**FIG 4:**
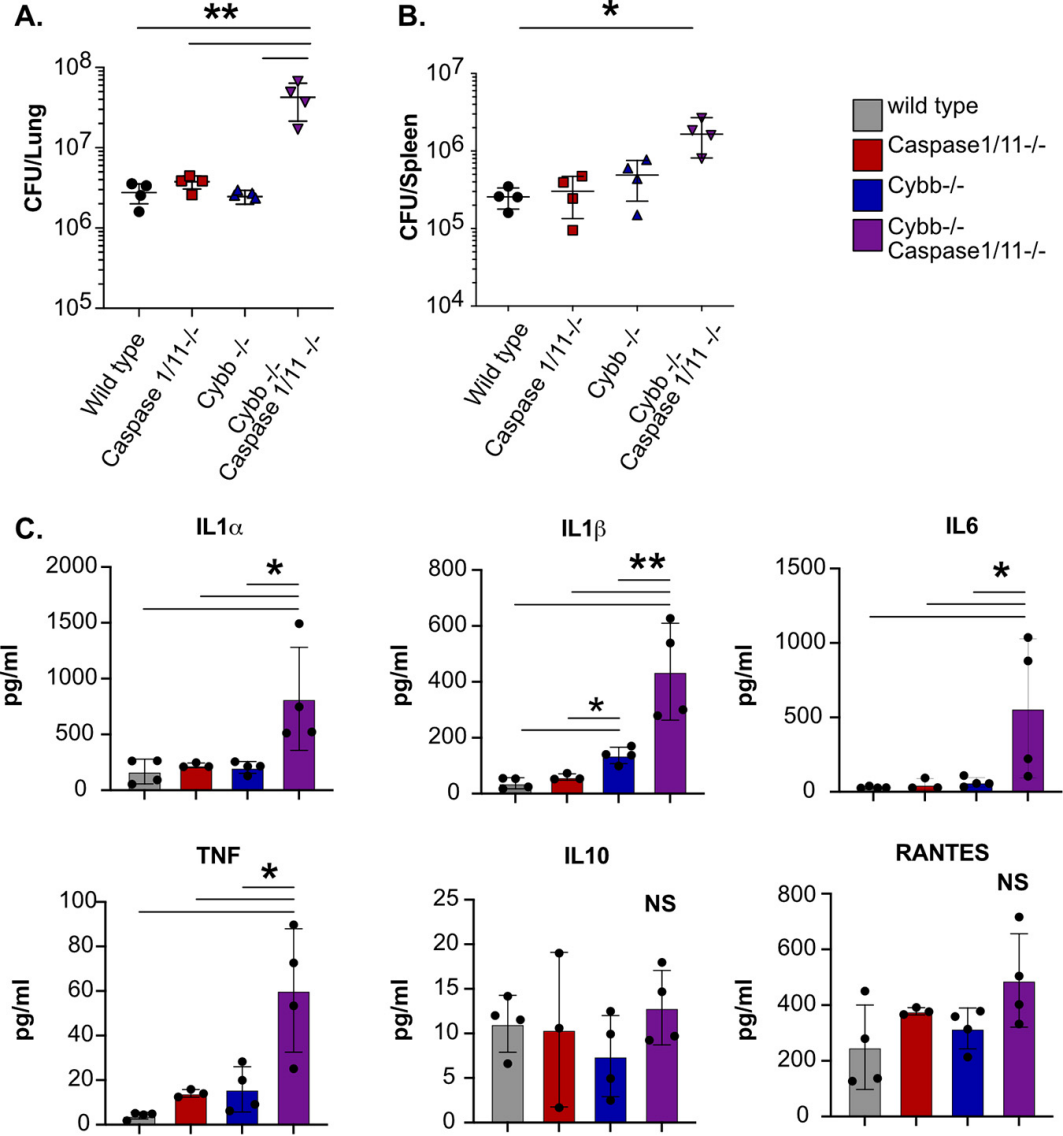
*Cybb^−/−^Caspase1/11^−/−^* mice do not control Mtb growth and are hyperinflammatory. Wild-type, *Caspase1/11^−/−^*, *Cybb^−/−^*, and *Cybb^−/−^Caspase1/11^−/−^* mice were infected with Mtb H37Rv YFP by the aerosol route in a single batch (day 1: 50 to 100 CFU). Lungs and spleen were collected at 25 days postinfection and used to quantify bacterial CFU. (A and B) Bacterial burdens in the lungs (A) and spleens (B) of mice. (C) Concentrations of cytokines in lung homogenates from infected mice were quantified (IL-1α, IL-1β, IL-6, TNF, IL-10, and RANTES). Each point represents a single mouse, data are representative of one experiment from three similar experiments. *, *P* < 0.05; **, *P* < 0.01; NS, no significance, by one-way ANOVA with a Tukey’s test for multiple comparisons. All comparisons between samples were made. No indicator of significance indicates that the comparison was deemed “not significant.”

We next sought to determine the mechanisms that drive the susceptibility of *Cybb^−/−^Caspase1/11^−/−^* animals. Wild type, *Cybb^−/−^*, *Caspase1/11^−/−^*, and *Cybb^−/−^Caspase1/11^−/−^* mice were infected with H37Rv YFP by low-dose aerosol, and 25 days later, viable Mtb in the lungs and spleen were quantified by CFU plating (27). We observed similar numbers of Mtb in wild-type, *Cybb^−/−^*, and *Caspase1/11^−/−^* animals in both organs, which is in line with previous reports (17, 20, 26). In contrast, over 10-fold more Mtb were present in the lungs and ~5-fold more Mtb were present in the spleens of infected *Cybb^−/−^Caspase1/11^−/−^* mice (Fig. 4A and B). We further characterized the cytokine profile from infected lung homogenates using a Luminex multiplex assay. We found that *Cybb^−/−^Caspase1/11^−/−^* mice expressed high levels of inflammatory cytokines, including IL-1α, IL-1β, TNF, and IL-6, but not IL-10 (Fig. 4C). We observed no significant differences between *Caspase1/11^−/−^* and wild-type mice, while in *Cybb^−/−^* mice we found increased levels of IL-1β but no other cytokines, in line with previous studies (17, 20, 26). Thus, mice deficient in both *Cybb* and *Caspase1/11* cannot effectively control Mtb replication and display hyperinflammatory cytokine responses.

### GR1^int^ CD11b^+^ granulocytes are recruited to the lungs of *Cybb^−/−^Caspase1/11^−/−^* mice during infection and are associated with Mtb.

**FIG 5:**
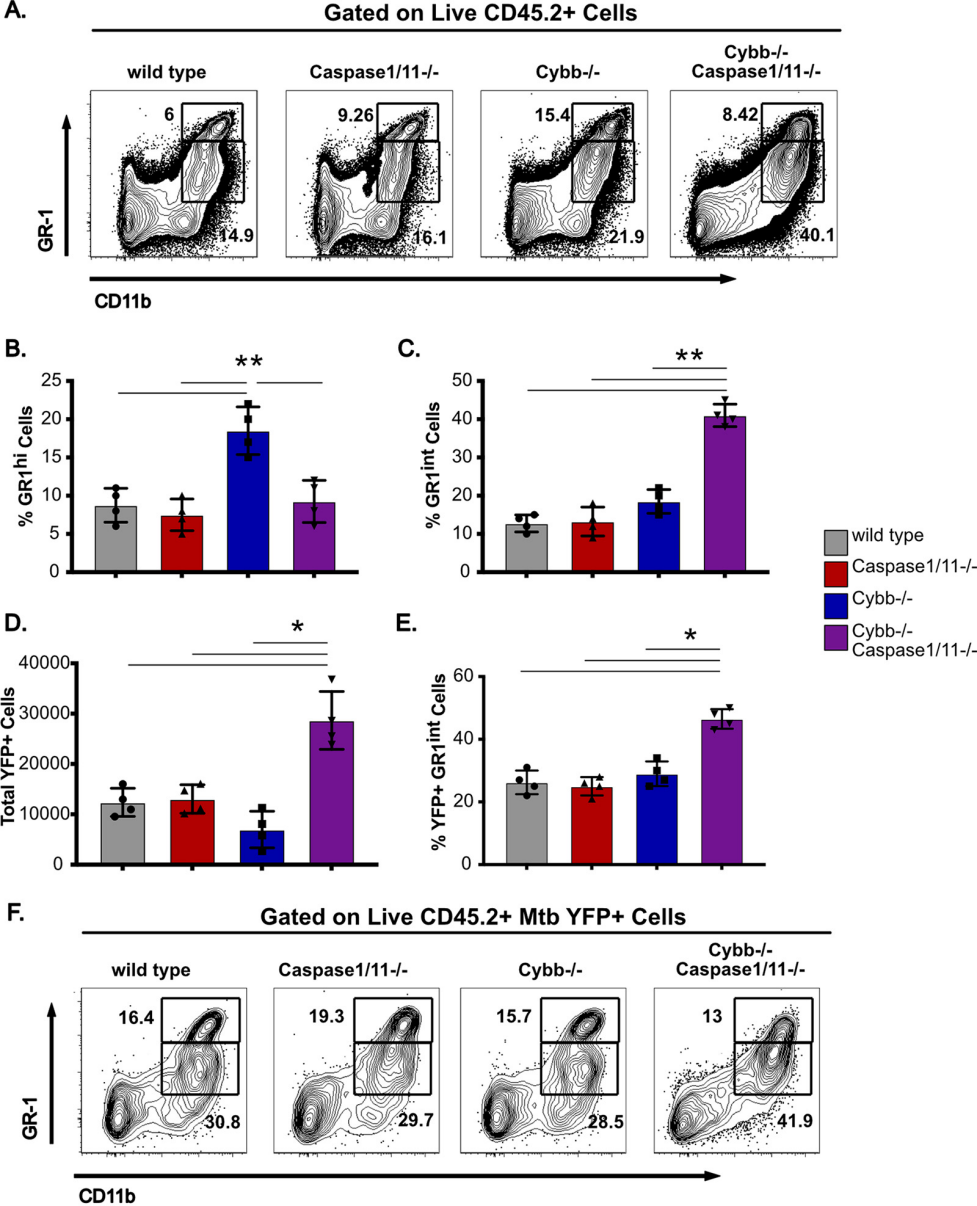
GR1^int^ granulocytes are recruited to the lungs and are associated with Mtb during infection of *Cybb^−/−^Caspase1/11^−/−^* mice. Wild-type, *Caspase1/11^−/−^*, *Cybb^−/−^*, and *Cybb^−/−^Caspase1/11^−/−^* mice were infected with Mtb H37Rv YFP by the aerosol route in a single batch (day 1: 50 to 100 CFU). Lungs were collected at 25 days postinfection, and single cell homogenates were made for flow cytometry analysis. (A) Representative flow cytometry plot of total lung granulocytes based on CD11b and GR1 staining (gated on live CD45.2^+^ single cells). Gates indicate CD11b^+^ GR1^hi^ or CD11b^+^ GR1^int^ granulocytes present in the lungs. (B) The percentages of gated cells (live CD45.2^+^ single cells) that were CD11b^+^ GR1^hi^ and (C) CD11b^+^ GR1^int^ were quantified. (D) Total number of H37Rv YFP^+^ cells was quantified from each mouse lung after gating on live, CD45.2^+^ single cells. (E) The percentage of gated H37Rv YFP^+^ cells that were CD11b^+^ GR1^int^ was quantified. (F) Representative flow cytometry plot of H37Rv YFP^+^-infected granulocytes (gated on live CD45.2^+^ YFP^+^ single cells). Gates indicate CD11b^+^ GR1^hi^ or CD11b^+^ GR1^int^ granulocytes present in the lungs. Each point represents a single mouse and data are representative of one experiment from three similar experiments. *, *P* < 0.05; **, *P* < 0.01 by one-way ANOVA with Tukey’s test for multiple comparisons.

The extreme susceptibility and increased Mtb growth observed in *Cybb*^−/−^*Caspase1/11*^−/−^ mice is similar to mice lacking *IFN-γ* or *Nos2* (28). Recent work showed that the susceptibility of *Nos2*–/– animals is driven by dysregulated inflammation that recruits permissive granulocytes to the lungs, which then allow amplified Mtb replication (29, 30). We hypothesized that similar responses might be associated with the susceptibility of *Cybb^−/−^Caspase1/11^−/−^* mice during Mtb infection. To test this hypothesis, we first analyzed the granulocyte populations of cells in the lungs of uninfected wild-type, *Caspase1/11^−/−^*, *Cybb^−/−^*, and *Cybb^−/−^Caspase1/11^−/−^* animals and observed no significant differences in the number of GR1^hi^ CD11b^+^ or GR1^int^ CD11b^+^ cells (Fig. S3). We then infected these animals with Mtb H37Rv YFP by low-dose aerosol for 25 days and analyzed all cells in the lungs by flow cytometry. We observed that infected wild-type and *Caspase1/11^−/−^* animals showed indistinguishable distributions of cells, and infected *Cybb^−/−^* mice recruited more GR1^hi^ CD11b^+^ neutrophils, in agreement with our previous findings (Fig. 5A and B) (17). However, we observed a significant increase in the total number of GR1^int^ CD11b^+^ cells in the lungs of infected *Cybb^−/−^Caspase1/11^−/−^* mice. This population is consistent with the permissive myeloid cells seen in mice that are highly susceptible to Mtb infection (29, 30).

If the recruited GR1^int^ CD11b^+^ granulocytes in the lungs of *Cybb^−/−^Caspase1/11^−/−^* mice are permissive for Mtb growth, we predicted that these cells would harbor a disproportionate fraction of intracellular Mtb in the lungs. To test this prediction, we quantified the total YFP^+^ infected cells from each genotype. We found an increase in the total number of YFP^+^ cells only in *Cybb^−/−^Caspase1/11^−/−^* mice (Fig. 5D). These data show that the lungs of *Cybb^−/−^Caspase1/11^−/−^* mice harbored more infected cells than the wild-type or single-knockout controls. We next examined the distinct cellular populations that were infected with Mtb in each genotype. We found that over 40% of infected cells in *Cybb^−/−^Caspase1/11^−/−^* mice were CD11b^+^GR1^int^ granulocytes, a significant increase compared to the wild-type, *Cybb^−/−^*, and *Caspase1/11^−/−^* animals (Fig. 5E and F). Together, these experiments show that Mtb infection of *Cybb^−/−^Caspase1/11^−/−^* mice is associated with the recruitment of granulocytes to the lungs, which harbor high levels of Mtb.

## DISCUSSION

While the phagocyte oxidase is undoubtedly important for protection against Mtb, the precise mechanisms by which it protects remain unclear (16, 17, 31, 32). In animal models, the loss of *Cybb* alone results in a loss of disease tolerance through increased Caspase1 activation (17). Our results show that the phagocyte oxidase also contributes to protection through a mechanism that is revealed only in the absence of Caspase1/11. While loss of either *Cybb* or *Caspase1/11* results in minor changes in survival, combining the mutations resulted in a dramatic increase in susceptibility, similar to mice lacking *IFN-γ*, *Nos2*, or *Atg5* (13, 14, 27, 28). The synthetic susceptibility phenotype was characterized by increased granulocyte influx and Mtb replication in the lungs. Whether this susceptibility is a result of failed antimicrobial resistance, failed tolerance, or both remains to be fully understood. However, based on the genetic interaction, it is likely that *Cybb* and *Caspase1/11* control parallel pathways that regulate cytokine and chemokine production and contribute to protection against TB.

While Mtb infection of both *Cybb^−/−^* and *Cybb^−/−^Casp1/11^−/−^* mice drives increased granulocyte trafficking to the lungs, the properties of these cells are distinct. In *Cybb^−/−^* mice, the granulocytes express high levels of GR1, and the distribution of Mtb-infected cells is unchanged compared to that in wild-type mice. In contrast, granulocytes recruited to the lungs of *Cybb^−/−^Caspase1/11^−/−^* mice express intermediate levels of GR1 and are associated with high levels of Mtb. A recent report characterizing the susceptibility of mice deficient in *Nos2* found that GR1^int^ granulocytes were long-lived, unable to control bacterial growth, and were not suppressive even with increased IL-10 production (29). In humans, low density granulocyte populations are associated with severe susceptibility to TB and may be analogous to these permissive GR1^int^ cells seen in susceptible mice (33). It is possible that these granulocytes do not directly drive susceptibility but rather are associated with uncontrolled TB disease caused by other defects in the host response. Future work using depletion and conditional knockouts will be required to understand how these changes in the cellular dynamics in *Cybb^−/−^Caspase1/11^−/−^* mice contribute to susceptibility.

Our current model predicts that the phagocyte oxidase and Caspase1/11 control the inflammatory state of myeloid cells during Mtb infection. When this control is lost, the result is a failure of disease tolerance, which drives progressive disease and recruits permissive granulocytes that modulate a feed-forward loop of inflammation, Mtb growth, and tissue damage. While the exact signals that recruit granulocytes to the lungs of *Cybb^−/−^Caspase1/11^−/−^* mice remain unclear, we observed dysregulation of IL-6, CXCL2, and IL-1α in Mtb-infected *Cybb^−/−^Caspase1/11^−/−^* macrophages. While the importance of each cytokine to *Cybb^−/−^Caspase1/11^−/−^* susceptibility needs to be examined extensively, IL-1α was the most significantly changed cytokine in *Cybb^−/−^Caspase1/11^−/−^* macrophages and *in vivo*. IL-1α is known to be required for protection against Mtb, as knockout mice are highly susceptible to disease (34). Whether exacerbated IL-1α directly contributes to TB susceptibility remains to be fully understood. Several non-mutually exclusive mechanisms could explain the dysregulation of IL-1α and possibly other cytokines. For example, there is evidence that changes in calcium influx and mitochondrial stability directly control the expression and processing of IL-1α (35). Thus, Cybb or Caspase1/11 may modulate calcium flux and mitochondrial function during Mtb infection, activating excessive IL-1α production. Recent studies have also suggested that metabolic pathways control ROS production that is directly required for the processing of GSDMD, which may link cellular metabolism to ROS signaling and inflammasome function (36). There is also evidence of a direct interaction between the phagocyte oxidase subunits and Caspase1 that modulates phagosome dynamics during Staphylococcus aureus infection, but whether this mechanism plays a role during Mtb infection remains unknown (37). Ongoing work is focused on examining the contribution of each potential mechanism to the susceptibility of *Cybb^−/−^Caspase1/11^−/−^* animals to better understand the regulatory networks that control inflammatory TB disease.

One outstanding line of questions from our findings is the specificity of the genetic interaction between *Cybb* and *Caspase1/11*. Both Caspase1- and Caspase11-dependent pathways are activated during Mtb infection, yet the direct contributions of either Caspase1 or Caspase11 to our observed susceptibility remains to be investigated by individually generating either *Cybb^−/−^Caspase1^−/−^* or *Cybb^−/−^Caspase11^−/−^* animals (38). Given the recent availability of clean *Caspase1* and *Caspase11* knockout mice, we are in the process of developing these models for the future (39). In addition, whether mutations in the inflammasome sensor NLRP3 or the adaptor ASC and other subunits of the phagocyte oxidase recapitulate the susceptibility of *Cybb^−/−^Caspase1/11^−/−^* remain unknown. Further dissecting these specific genetic interactions between other phagocyte oxidase and inflammasome components will help elucidate the underlying mechanisms that control the susceptibility observed in *Cybb^−/−^Caspase1/11^−/−^* animals.

Our discovery of a synthetic susceptibility to Mtb in *Cybb^−/−^Caspase1/11^−/−^* mice was serendipitous. Because the susceptibility observed in *Cybb^−/−^* mice was found to be due to dysregulated Caspase1 activation and IL-1β production, we initially hypothesized that the combined loss of *Cybb* and *Caspase1* would reverse the tolerance defects found in mice lacking the phagocyte oxidase, and were surprised to uncover a synthetic susceptibility. Since phagocyte oxidase and inflammasomes are among the most studied pathways in immunology, our findings highlight a fundamental lack of understanding of interactions between immune signaling networks that control inflammation and immunity. To develop new host-directed therapeutics that could shorten treatment times and improve disease control, it is critical to understand how these interconnected networks function to protect against TB. A major obstacle in identifying protective networks against Mtb is the redundancy among host pathways, which masks important functions in single-knockout animals. A global understanding of the genetic interactions that impact key inflammatory networks during TB would significantly inform the development of effective host-directed therapies or immunization strategies. Large-scale genetic interaction studies are common in cancer biology and should be applied to immune signaling networks during Mtb infection to better define these critical but currently unknown mechanisms that control protection against TB (40). Together, these findings suggest that genetic interactions are key regulators of protection against Mtb, with Cybb and Caspase1/11 contributing together to protect against TB.

## MATERIALS AND METHODS

### Mice and ethics statement.

Mouse studies were performed in accordance using the recommendations from the Guide for the Care and Use of Laboratory Animals of the National Institutes of Health and the Office of Laboratory Animal Welfare. Mouse studies were performed using protocols approved by the Institutional Animal Care and Use Committee (IACUC) in a manner designed to minimize pain and suffering in Mtb-infected animals. All mice were monitored and weighed regularly. Mice were euthanized following an evaluation of clinical signs with a score of 14 or higher. C57BL6/J mice (no. 000664) and *Cybb^−/−^* mice (no. 002365) were purchased from Jackson Labs. *Caspase1/11^−/−^* mice were a kind gift from Katharine Fitzgerald and *Cybb^−/−^Caspase1/11^−/−^* mice were generated in-house. All mice were housed and bred under specific pathogen-free conditions and in accordance with the University of Massachusetts Medical School (Sassetti Lab A221-20-11) and Michigan State University (PROTO202200127) IACUC guidelines. All animals used for experiments were 6 to 12 weeks old.

### Macrophage and dendritic cell generation.

Bone-marrow derived macrophages and dendritic cells were obtained from the femurs and tibias of sex- and age-matched mice. For BMDMs, cells were cultured in 10 cm^2^ non-tissue culture-treated petri dishes with 10 mL Dulbecco’s modified Eagle medium (DMEM) with 10% fetal bovine serum (FBS) and 20% L929 supernatant for 1 week. On day 3, the old medium was decanted and fresh differentiation medium was added. After 7 days of differentiation, cells were lifted in phosphate-buffered saline (PBS) with 10 mM EDTA, seeded in tissue-culture treated dishes in DMEM with 10% FBS with no antibiotics, then used the following day for experiments.

For BMDCs, cells were cultured in 10cm^2^ non-tissue culture treated petri dishes with 10 mL DMEM with 10% FBS, l-glutamine, 2 μM 2-mercaptoethanol, and 10% supernatant from B16-GM-CSF cells as described previously. After 7 days of differentiation, BMDCs were further enriched by isolating loosely adherent cells, removing F4/80^+^ cells, and then isolating CD11c^+^ cells by bead purification following the manufacturer’s instructions (Stem Cell Tech). Cells were then plated in tissue culture-treated dishes in DMEM with 10% FBS and used the following day for experiments.

### Bone marrow-derived macrophage and dendritic cell infections and analysis.

Phthiocerol dimycocerosates (PDIM)-positive H37Rv was grown in 7H9 medium containing 10% oleic albumin dextrose catalase growth supplement and 0.05% Tween 80 as done previously (17). Prior to infection, cultures were washed in a PBS-0.05% Tween solution and resuspended in DMEM with 10% FBS. To obtain single cell suspensions, samples were centrifuged at 200 × *g* for 5 min to remove clumps. Culture density was determined by taking the supernatant from this centrifugation and determining the optical density at 600 nm (OD_600_), with the assumption that OD_600_ = 1.0 is equivalent to 3 × 10^8^ bacteria per mL. Bacteria were added to macrophages for 4 h, then cells were washed with PBS and fresh medium was added. For cytokine analysis, at the indicated time points, supernatants were harvested and centrifuged through a 0.2-μm filter. Supernatants were then analyzed by a Luminex multiplex assay (Eve Technology) or by ELISA following manufacturer protocols (R&D Systems). For CFU analysis, at the indicated time points, 1% saponin was added to each well without removing the medium to lyse cells while maintaining extracellular bacteria. Serial dilutions were then completed in PBS containing Tween 80 (PBS-T) and dilutions were plated on 7H10 agar. For cell death experiments, at the indicated time points, medium was removed and a LDH assay (Promega) was completed following the manufacturer’s instructions.

### Mouse infections and CFU quantification.

For animal infections, H37Rv or YFP^+^ H37Rv was resuspended in PBS-T. Prior to infection, bacteria were sonicated for 30 s, then delivered into the respiratory tract using an aerosol generation device (Glas-Col). To verify low-dose aerosol delivery, a subset of control mice was euthanized the following day. Otherwise, the endpoints are designated in the figure legends. To determine total CFU in either the lung or spleen, mice were anesthetized via carbon dioxide asphyxiation and cervical dislocation, the organs were removed aseptically and homogenized, and 10-fold serial dilutions of each organ homogenate were made in PBS-T, plated on 7H10 agar plates, and incubated at 37°C for 21 to 28 days. Viable bacteria were then counted. Both male and female mice were used throughout the study and no significant differences in phenotypes were observed between sexes.

### Flow cytometry.

Analysis of infected myeloid cells in the lungs was performed as previously described (10, 29). In short, lung tissue was homogenized in DMEM containing FBS using C-tubes (Miltenyi Biotec). Collagenase type IV/DNase I (Sigma-Aldrich) was added, and tissues were dissociated for 10 s on a GentleMACS system (Miltenyi Biotec). Lung tissue was then oscillated for 30 min at 37C. Following incubation, tissue was further dissociated for 30 s on a GentleMACS. Single cell suspensions were isolated following passage through a 40-μm filter. Cell suspensions were then washed in DMEM and aliquoted into 96-well plates for flow cytometry staining. Non-specific antibody binding was first blocked using Fc-Block. Cells were then stained with anti-GR1 Pacific Blue, anti-CD11b PE, anti-CD11c APC, and anti-CD45.2 PercP Cy5.5 (BioLegend). Live cells were identified using Zombie Aqua (BioLegend). No antibodies were used in the FITC (fluorescein isothiocyanate) channel to allow quantification of YFP^+^ Mtb in the tissues. All experiments contained a non-fluorescent H37Rv infection control to identify infected cells. Cells were stained for 30 min at room temperature and fixed in 1% paraformaldehyde for 60 min. All flow cytometry was run on a MACSQuant Analyzer 10 (Miltenyi Biotec) or an Attune Cytpix and was analyzed using FlowJo version 10 (Tree Star).

### Statistical analysis and data availability.

Statistical analyses were performed using Prism 10 (GraphPad) software as described previously (17, 41). The statistical tests used for each experiment are described in each figure legend, along with symbols indicating significance or no significance. All data shown, unless indicated otherwise, are representative of one of several experimental replicates. Data from all replicate experiments for each figure are available for download at the Olive Research Group GitHub (https://github.com/OliveLab-MSU).
