# Supplementary figures and images for: High Lethality of Mycobacterium tuberculosis Infection in Mice Lacking the Phagocyte Oxidase and Caspase1/11
Source: Infect Immun. 2023 Jun 14;91(7):e00060-23. doi: 10.1128/iai.00060-23 (PMC10353354; doi:10.1128/iai.00060-23)

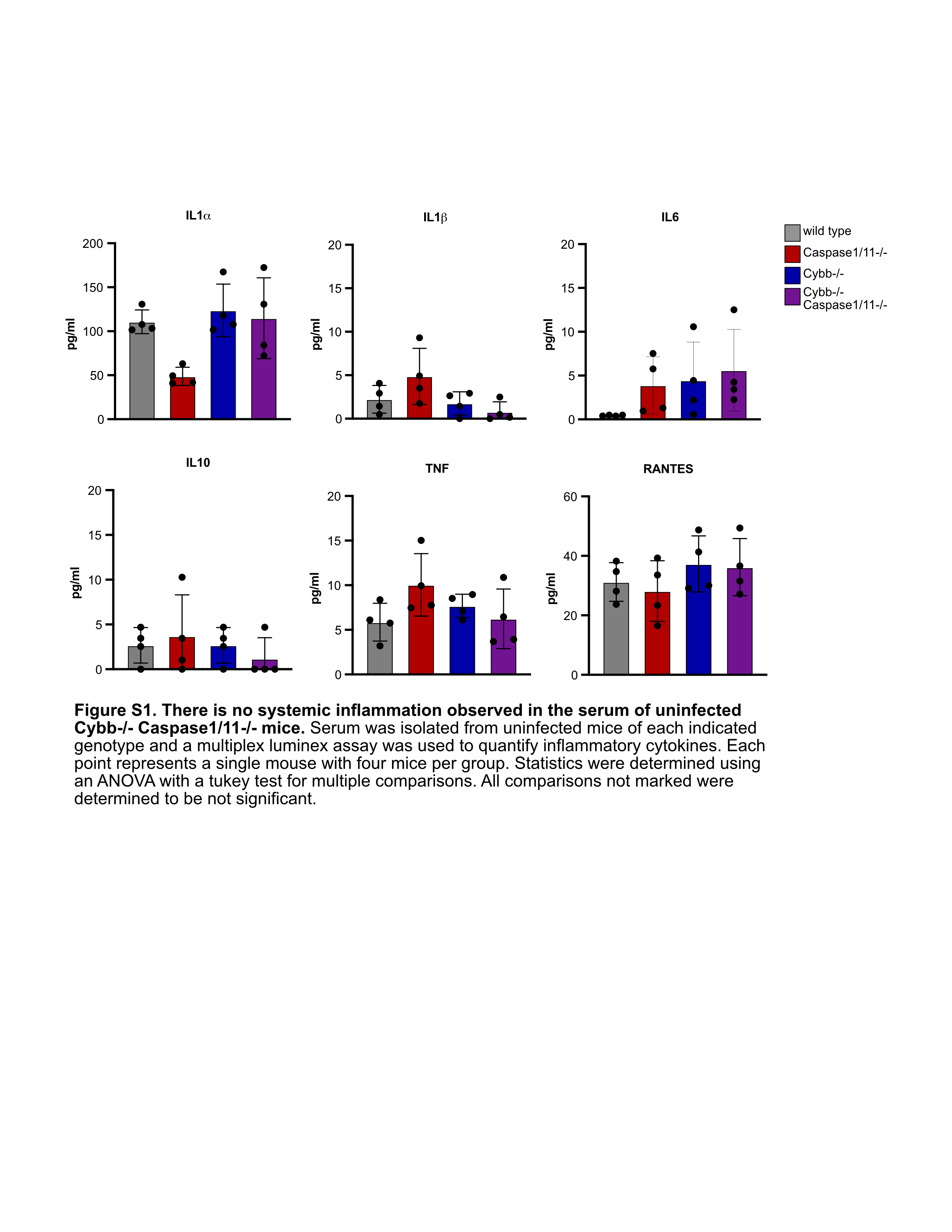

Supplement: Supplemental file 1 — Fig. S1. Download iai.00060-23-s0001.tif, TIF file, 0.7 MB [file iai.00060-23-s0001.tif]

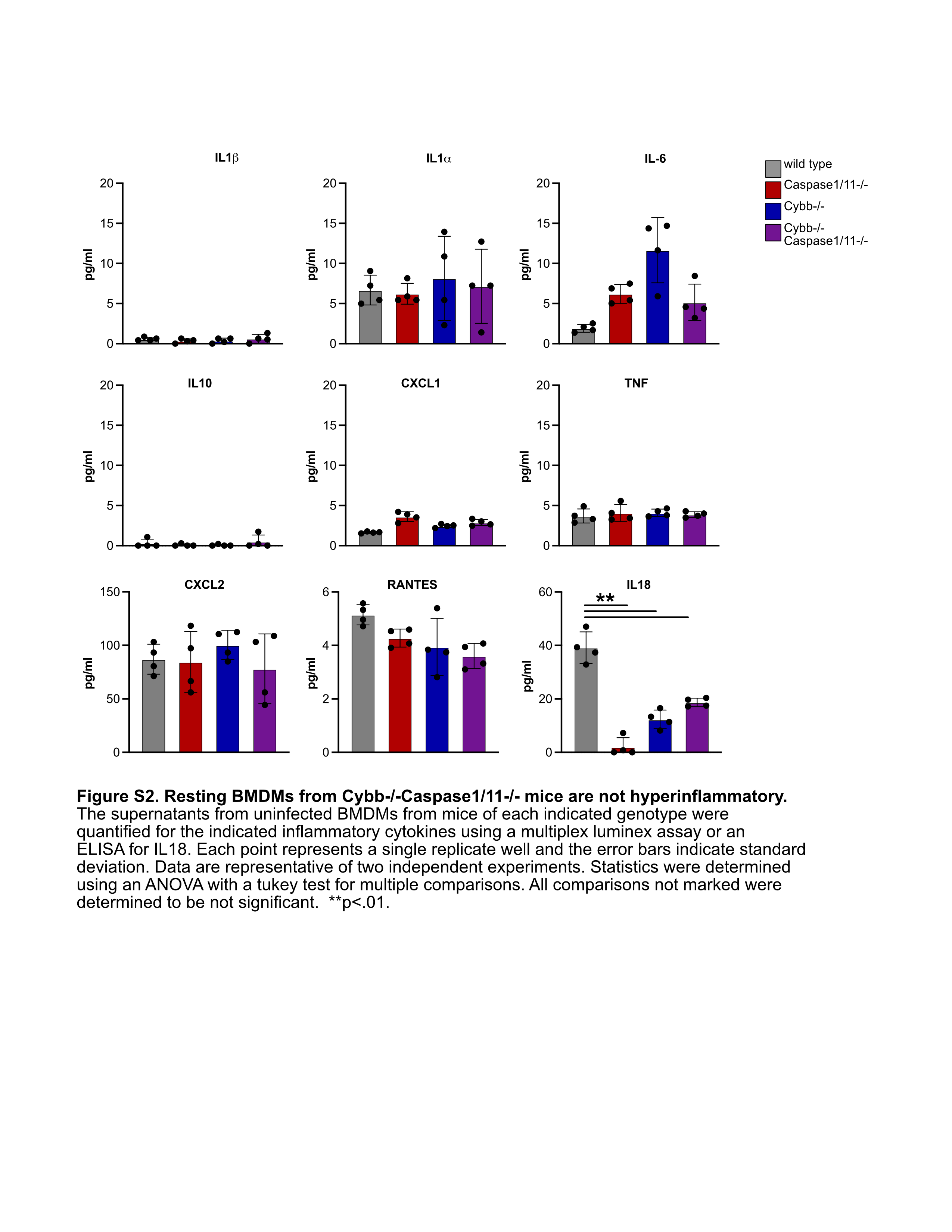

Supplement: Supplemental file 2 — Fig. S2. Download iai.00060-23-s0002.tif, TIF file, 0.8 MB [file iai.00060-23-s0002.tif]

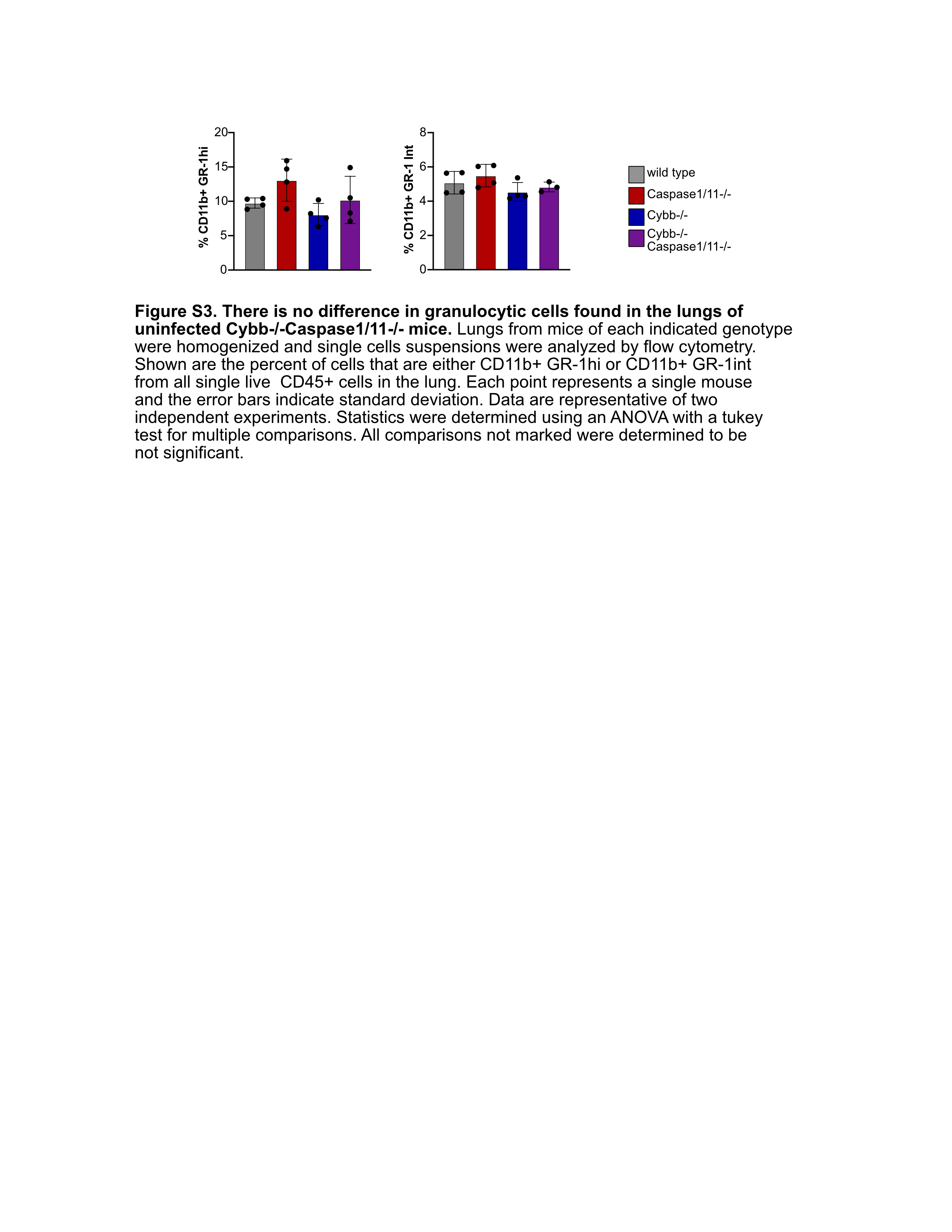

Supplement: Supplemental file 3 — Fig. S3. Download iai.00060-23-s0003.tif, TIF file, 0.6 MB [file iai.00060-23-s0003.tif]
